# Impact of Dihedral Angle in Conjugated Organic Cation on the Structures and Properties of Organic‐Inorganic Lead Iodides

**DOI:** 10.1002/chem.202402535

**Published:** 2024-12-04

**Authors:** Bidhan Chandra Patra, Ruichen Wan, Curtis E. Moore, Yiying Wu

**Affiliations:** ^1^ Department of Chemistry and Biochemistry The Ohio State University Columbus, Ohio 43210 United States

**Keywords:** Dihedral angle, 2,2’-Bipyridine, HOIMHs (hybrid organic-inorganic metal halides), One-dimensional (1D) crystals, Band gap tuning

## Abstract

Conjugated organic cations are intriguing for organic‐inorganic halide perovskites due to their direct participation in the optoelectronic properties of hybrid materials. In conjugated cations, the dihedral angle, or torsion angle, between adjacent aromatic rings is a critical secondary structural element. This angle influences not only the shape of the cations but also the overlap between the π‐orbitals on adjacent rings, thereby affecting their electronic properties. Understanding how variations in the dihedral angle impact the structure and properties of hybrid organic‐inorganic metal halides (HOIMHs) is fundamentally important. In this study, we utilized 2,2′‐dimethyl bipyridinium as the organic cation, reacting it with PbI₂ to form hybrid lead iodides. Remarkably, variations in the dihedral angle between the two pyridinium rings resulted in the formation of two distinct crystal structures with different band gaps. Our findings demonstrate that manipulating the dihedral angle offers a novel approach to controlling the structures and properties of hybrid metal halides with conjugated cations.

## Graphical abstract

Dihedral angle inside the organic cation significantly change the crystal packing as‐well‐as optical property of hybrid organic‐inorganic metal halides (HOIMHs). We used N‐substituted 2‐2’‐bipyridinium cation (iodide counter‐ion) for the synthesis of HOIMHs. The change in dihedral angle of the organic cation gives two different hybrid crystal with different optical property.

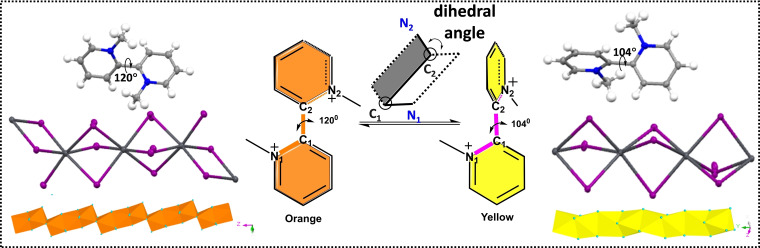

## Introduction

Organic cations play a vital role in determining the structures and properties of hybrid organic‐inorganic metal halides (HOIMHs). Their size and shape directly influence the dimension of hybrid materials including three dimension (3D), two dimension (2D), one dimension (1D) and zero dimension (0D).[^1^, ^2^] Tolerance factor (t) is used to predict the structural stability of 3D perovskites (ABX_3_).[^3^, ^4^, ^5^] For the formation of low‐dimensional perovskites, factors such as the size and shape of cations, especially the steric hindrance around nitrogen atoms, are important.[^6^, ^7^, ^8^, ^9^] Our group reported a machine learning (ML)‐assisted approach to predict the dimensionality of lead iodide‐based perovskites.^[10]^ Four structural features, including steric effect index, eccentricity, largest ring size, and hydrogen‐bond donor, have been identified as the key controlling factors. More recently, our group has pointed out the importance of considering the secondary structure of organic cations when they are packed in hybrid crystals, and used a series of alkoxy‐ammoniums as the model system to investigate the influence of intramolecular hydrogen bonding on the secondary structure of organic cations, which change their shape and steric hindrance, and the resultant crystal structure of hybrid materials.^[11]^

Among the vast array of organic cations, conjugated ones are of particular interest due to their ability to integrate the electronic and optical properties of organic semiconductors into hybrid materials. For instance, Mitzi et al. pioneered the field by incorporating the first functional quater‐thiophene molecule (AEQT) into an inorganic perovskite framework to form 2D perovskites.[^12^, ^13^] Similarly, Braun et al. incorporated pyrene molecules into a mixed‐halide inorganic framework as chromophores.^[14]^ More recently, the Dou group have designed and synthesized a series of new thiophene‐based monovalent cations and other conjugated cations, which were successfully incorporated into the 2D halide perovskite framework and were demonstrated to have excellent performance in a variety of electronic and optoelectronic devices.[^15^, ^16^] Beyond 2D structures, viologen‐based cations have been integrated into 1D HOIMHs.[^17^, ^18^, ^19^, ^20^, ^21^] The low‐lying π* orbitals in viologen derivatives form the conduction band edge of these 1D semiconductors, reducing their bandgap for efficient absorption of visible light. The stable photoelectrochemistry of methyl viologen in polar solvents has also been demonstrated.^[20]^ But the study on dihedral angle dependent structural change in hybrid materials are yet to be explored.

In conjugated cations, the dihedral angle, also known as the torsion angle, between adjacent aromatic rings represents a crucial secondary structural element. This angle not only influences the shape of the cations but also significantly impacts the overlap between the π orbitals on adjacent rings, resulting in different electronic properties.^[22]^ Therefore, it is fundamentally important to investigate how variations in the dihedral angle affect the structure and properties of HOIMHs. In this study, we used 2,2′‐dimethyl bipyridinium as the organic cation to react with PbI₂, forming hybrid lead iodides. Interestingly, two distinct crystal structures were obtained from variation of the dihedral angle between the two pyridinium rings. This study shows that dihedral angle may provide a novel approach for controlling the structures and properties of hybrid metal halides with conjugated cations.

## Results and Discussion

Herein, we have reported a 2‐2’‐bipyridinium based 1D HOIMH named as 2‐2’‐DMBPyPbI, where the organic salt 2‐2’‐dimethyl bipyridinium di‐iodide (2‐2’‐DMBPyI_2_) plays a crucial role for the structural variation of the hybrid material. 2‐2’‐DMBPyPbI was synthesized according to the previous literature report (see the detailed synthetic procedure in the supporting information file).^[21]^

**Figure 1:**
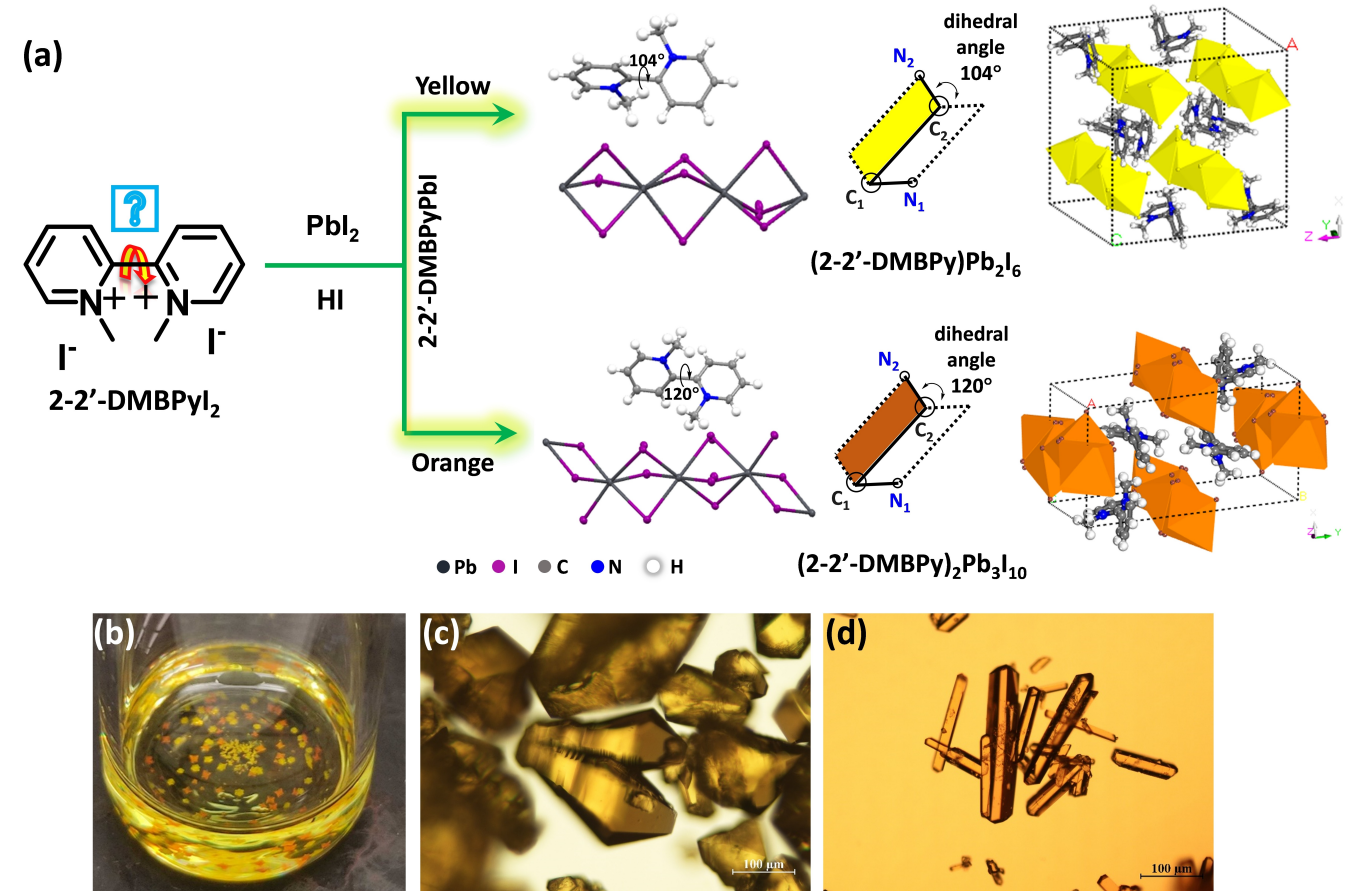
(a) Schematic presentation for the formation of 2‐2’‐DMBPyPbI; (b) Photographs yellow and orange crystals that were grown in same environment. (c, d) optical microscope images of yellow [2‐2’‐DMBPy)Pb_2_I_6_] and orange [2‐2’‐DMBPy)_2_Pb_3_I_10_] crystals. The yellow and orange crystals possess octahedral and rod like morphology respectively.

Initially, 2‐2’‐DMBPyI_2_ was synthesized by reaction between 2‐2’‐bipyridine (BPy) and methyl iodide (MeI). Then 2‐2’‐DMBPyI_2_ was mixed with 57 % HI solution of lead iodide (PbI_2_) and the solution was heated at 100 °C for complete solubilization. It was kept for few minutes for cooling and both the orange and yellow crystals were grown inside the same solution (Figure 1a and b). Those crystals were mounted for SCXRD study (Table 1). Single crystal x‐ray diffraction (SCXRD) study indicates that the yellow and orange crystals were crystalized in monoclinic (Figure 1c) and orthorhombic (Figure 1d) crystal system respectively. Furthermore, it was observed that the dihedral angle of the two pyridinium rings in the orange and yellow crystals were 120° and 104° respectively. From our previous study of methylviologen Lead Iodide, we noted that the dihedral angle between the two pyridinium rings reside nearly 180° and the color tends to deep red.^[20]^ In our present study, we observed that as the central dihedral angle gradually decreased, the color of the materials changed significantly, along with the crystal packing. The decrease of dihedral angle indicates that the two pyridinium units come in different planes, which hindered the conjugation inside the bipyridinium, leading to the color change from orange to yellow.

**Table 1:** Refinement Parameters of (2‐2’‐DMBPy)_2_Pb_3_I_10_ and (2‐2’‐DMBPy)Pb_2_I_6_.

Compounds	(2‐2’‐DMBPy)_2_Pb_3_I_10_ (Orange Crystal)	(2‐2’‐DMBPy)Pb_2_I_6_ (Yellow Crystal)
CCDC number	2351399	2351398
Chemical formula	(C_12_ H_14_ N_2_)_2_ Pb_3_ I_10_	(C_12_ H_14_ N_2_) Pb_2_ I_6_
Temperature	100.0 K	100.0 K
Crystal system	Monoclinic	Orthorhombic
Space group	P 2_1_/n	Pbca
*a*	11.0901(3) Å	17.5748(4) Å
*b*	17.2367(5) Å	16.0015(3) Å
*c*	12.4168(3) Å	18.0671(3) Å
*α*	90°	90°
*β*	112.8130(10)°	90°
*γ*	90°	90°
Volume	2187.88(10) Å^3^	5080.89(17) Å^3^
Z	2	8
Density (calculated)	3.435 mg/m^3^	3.561 mg/m^3^

**Figure 2:**
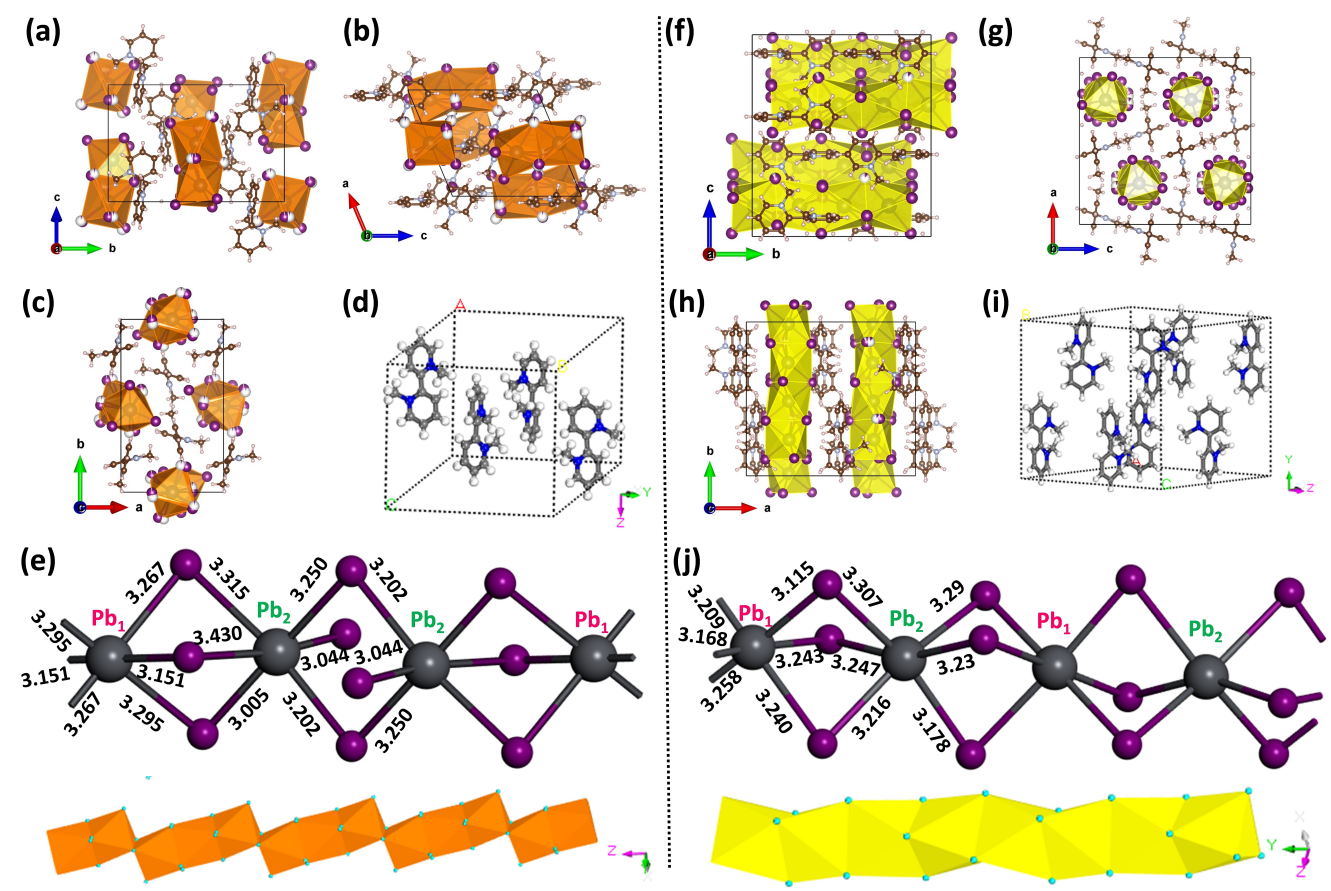
(a, b, c) Crystal packing of (2‐2’‐DMBPy)_2_Pb_3_I_10_ viewed in ‘a’, ‘b’ and ‘c’ direction respectively. (C, N, H, Pb and I were marked as brown, sky blue, baby pink, dark grey and purple color balls respectively). (d) Packing of organic cations inside one unit cell of (2‐2’‐DMBPy)_2_Pb_3_I_10_ (orange crystal). (e) Two types of co‐ordination environment involving [PbI_6_] octahedron of (2‐2’‐DMBPy)_2_Pb_3_I_10_ and the I−Pb−I bond length (in Å) and the packing of inorganic chain. (f, g, h) Crystal packing of (2‐2’‐DMBPy) Pb_2_I_6_ viewed in ‘a’, ‘b’ and ‘c’ direction respectively. (C, N, H, Pb and I were marked as brown, sky blue, baby pink, dark grey and purple color balls respectively) (i) Packing of organic cations inside one unit cell of (2‐2’‐DMBPy)Pb_2_I_6_ (yellow crystal). (j) Two types of co‐ordination environment involving [PbI_6_] octahedron of (2‐2’‐DMBPy) Pb_2_I_6_ and the I−Pb−I bond length (in Å) and packing of inorganic chain.

Figure 2a–d shows the orange crystal structure with stoichiometric ratio of (2‐2’‐DMBPy)_2_Pb_3_I_10_. The (2‐2’‐DMBPy)_2_Pb_3_I_10_ crystalized in the monoclinic crystal system with P 2_1_/n space group. The inorganic Pb−I octahedral building blocks form a 1D chain inside the 2‐2’‐DMBPy^2+^ cation. Those octahedrons are connected via both face sharing and edge sharing to construct the 1D chain. Figure 2f–i shows the crystal structure of the yellow crystal with the stoichiometry ratio (2‐2’‐DMBPy)Pb_2_I_6_ where each Pb^2+^ is connected by three tri‐coordinate center iodides through the face sharing surrounded by 2‐2’‐DMBPy^2+^ cation. The (2‐2’‐DMBPy)Pb_2_I_6_ crystalized in the orthorhombic crystal system with *Pbca* space group, forming an infinite inorganic chain of 1D structure.

It is usually quantitatively evaluated and compared by the mean square relative deviation of the octahedral bond length Δ_oct_ (bond length distortion) and octahedral bond angle distortion variance σ^2^_oct_ (Figure 2e and j).^[21]^ It can be calculated by the following equation:

(1) $$\Delta_{oct}=\frac{1}{6}\sum_{i=1}^{6}\frac{\left|d_{i}-d_{0}\right|}{d_{0}}$$

(2)

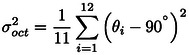




where ***d***_***i***_, ***d***_***0***_ and ***θ***_***i***_ are the individual metal‐halide bond length, the average bond length and the individual halide‐metal‐halide bond angle in a single octahedron, respectively. The bond length of (2‐2’‐DMBPy)_2_Pb_3_I_10_ ranges from 3.151 Å to 3.298 Å and the bond length of (2‐2’‐DMBPy)Pb_2_I_6_ ranges from 3.115 Å to 3.258 Å. The ***Δ***_***oct***_ and ***σ*** ^***2***^_***oct***_ of the two different [PbI_6_] octahedra in (2‐2’‐DMBPy)_2_Pb_3_I_10_ and (2‐2’‐DMBPy)Pb_2_I_6_ is mentioned in Table 2. For an ideal octahedron, the ***Δ***_***oct***_ and ***σ*** ^***2***^_***oct***_ should be zero and any deviation i. e. higher in values causes higher distortion inside the crystal.[^23^, ^24^] The octahedral distortion was calculated from the refined crystal structure as shown in Table 2. The bond length distortion (**Δ_oct_**) is similar in both the crystals but the bond angle variance parameters (**σ^2^**_**oct**_) differ significantly, indicates that the structural distortion mostly comes from the I−Pb−I bond angles variance.^[25]^

**Table 2:** Crystal disorder parameters of (2‐2’‐DMBPy)_2_Pb_3_I_10_ and (2‐2’‐DMBPy)Pb_2_I_6_.

Compounds	*Δ_oct_ of [PbI_6_]*		*σ* ^*2*^_*oct*_ *of [PbI_6_]*	
	^[#]^ *Pb_1_*	^[#]^ *Pb_2_*	^[#]^ *Pb_1_*	^[#]^ *Pb_2_*
(2‐2’‐DMBPy)_2_Pb_3_I_10_	0.01785	0.0386	61.95	36.11
(2‐2’‐DMBPy)Pb_2_I_6_	0.0133	0.0113	19.48	144.14

[#] Two different ‘Pb’ environments inside the crystal (Figure 2e and j).

To prove the relative stability between the yellow and orange crystals, we have kept both the crystals inside the precursor solution for one week. It was observed that all the yellow crystals were turned into orange crystals. This indicates that the orange crystals are the thermodynamically stable product where the dihedral angle of the organic cation (2‐2’‐DMBPy^2+^) is higher (120°) compared to the organic cation in the yellow crystals.

**Figure 3:**
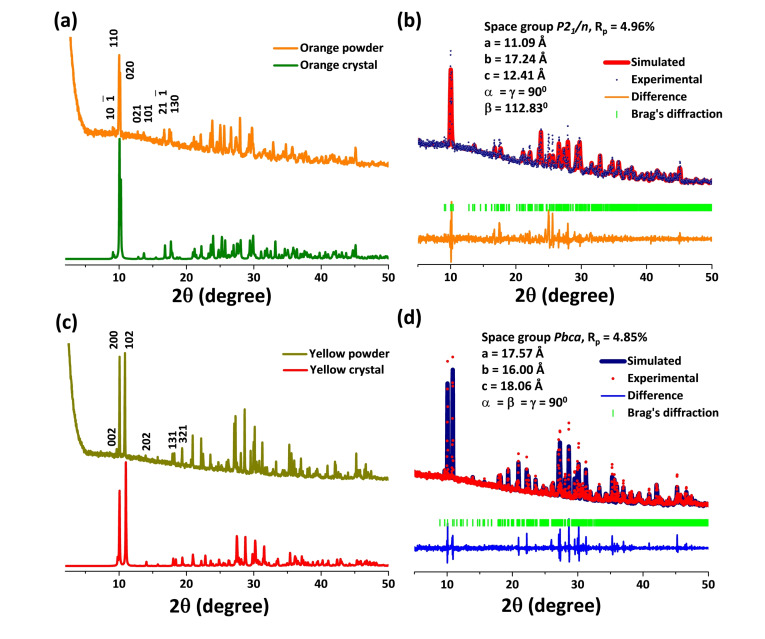
(a) PXRD pattern of (2‐2’‐DMBPy)_2_Pb_3_I_10_ powder (orange color line) in comparison with XRD parent obtained form (2‐2’‐DMBPy)_2_Pb_3_I_10_ crystal (dark green color line). (b) Pawley refinement of the PXRD pattern of (2‐2’‐DMBPy)_2_Pb_3_I_10_ with the corresponding crystal. (c) PXRD pattern of (2‐2’‐DMBPy) Pb_2_I_6_ powder (dark yellow color line) in comparison with XRD parent obtained form (2‐2’‐DMBPy)Pb_2_I_6_ crystal (red color line). (d) Pawley refinement of the PXRD pattern of (2‐2’‐DMBPy)Pb_2_I_6_ with the corresponding crystal.

We have also synthesized orange powder by reaction between the PbI_2_ solution with 2‐2’‐DMBPyI_2_ salt at room temperature. 0.5 (mmol) PbI_2_ was dissolved in 2 ml hydro‐iodic acid (HI) under boiling condition. The solution was then kept for cooling and the 2‐2’‐DMBPyI_2_ was added to the solution at room temperature. A deep orange color powder was formed which was washed with acetonitrile and used for further study. From the PXRD analysis, it was observed that both the orange powder and crystal was crystalized in the same phase. The first two intense peak at 2θ values of 10.06° and 10.26° indicates the (110) and (020) planes respectively (Figure 3a). Further, the Pawley refinement was carried out which clearly indicates the similar phase purity between powder and crystal with the R_p_ factor of 4.96 % (Figure 3b).

Yellow powder was synthesized by controlled heating of PbI_2_ solution with 2‐2’‐DMBPyI_2_. Initially PbI_2_ was dissolved in HI solution under heating condition. 2‐2’‐DMBPyI_2_ was added to it and keep boiling until all the orange powder turns yellow. The powder was washed with acetonitrile for further characterization. PXRD analysis shows that the yellow powder contains two intense peaks at 2θ values of 10.06° and 10.99° for (200) and (102) planes respectively, with other additional peaks (Figure 3c). These peaks were further simulated by the Pawley refinement with the XRD pattern obtained from the yellow crystals with the R_p_ factor of 4.85 % (Figure 3d). It was observed that both the yellow powder and crystal were crystalized in the identical planes with similar phase purity.

**Figure 4:**
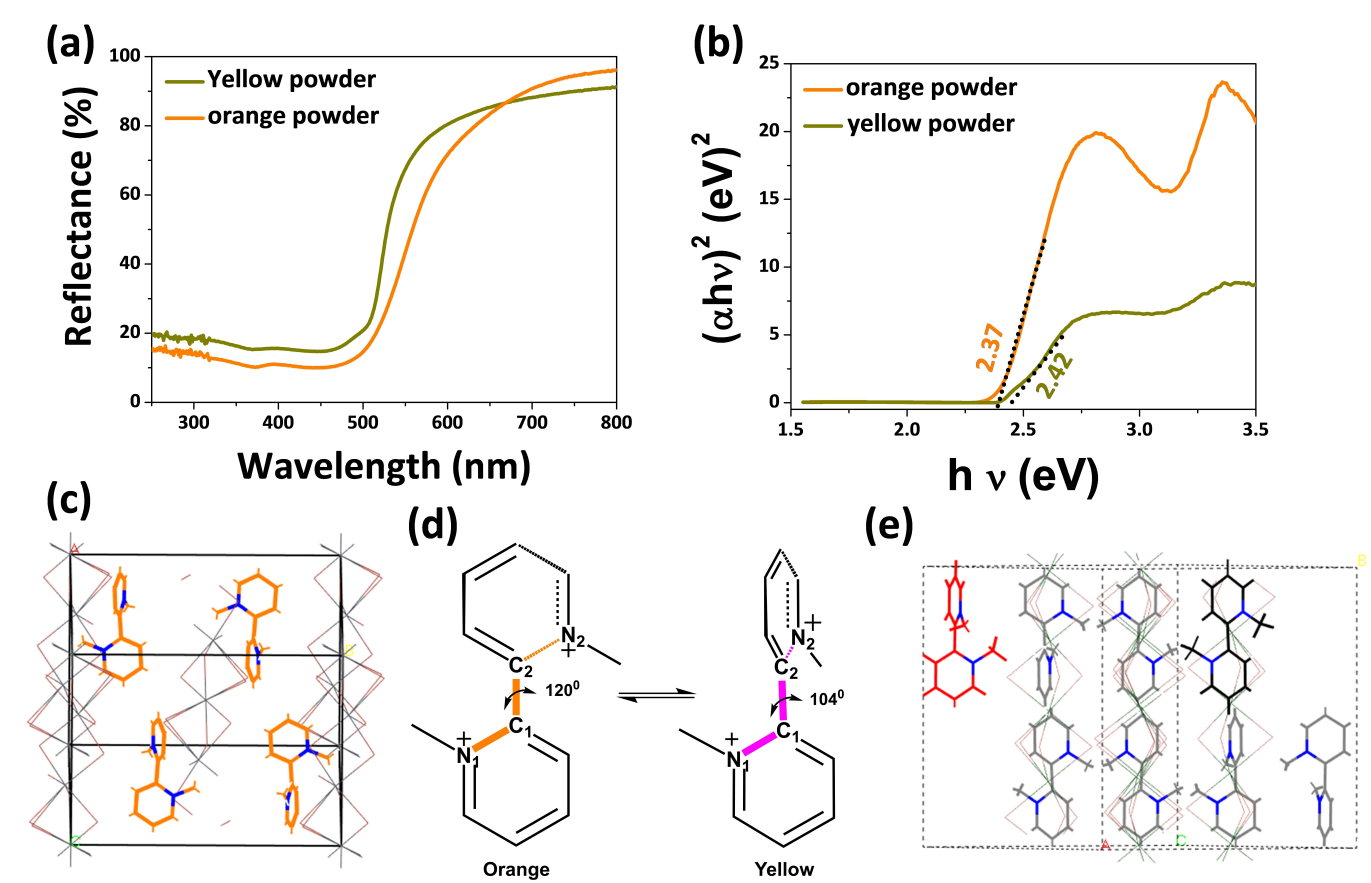
(a) Diffuse reflectance spectroscopy of orange powder [2‐2’‐DMBPy)_2_Pb_3_I_10_] and yellow powder (2‐2’‐DMBPy)Pb_2_I_6_. (b) Band gap between the orange and yellow powder. (c, e) crystal packing of orange and yellow crystal respectively where the central dihedral angle between the two pyridine rings are 120° and 104° respectively. The ‘Pb’ and ‘I’ bonds were blurred intentionally for better clarity of organic ligands inside the network. (d) Schematic presentation of the ring flipping by change in dihedral angle between the two bipyridine ring inside the orange and yellow crystals. (N_1_−C_1_−C_2_ and C_1_−C_2_−N_2_ forms two different planes i. e. dihedral angle). C_1_−C_2_ bond length: (orange 1.496 Å, yellow 1.497 Å), indicating single bond nature.

It is well established that the dihedral angle controls the conjugation inside the organic molecule. Increasing the dihedral angle causes the lowering of band gap due to the smooth conjugation between the p‐orbitals (participating in conjugation), which are in same plane. But upon decreasing the dihedral angle, the *p*‐orbitals comes in different planes, causes the hindrance in conjugation.^[26]^ Now, depending on the ligand substitution, this dihedral angle can be tuned inside the HOIMHs which leads to the formation of variable structure and different symmetry.[^27^, ^28^, ^29^] Using this concept, we observed dihedral angle dependent optical property between the orange and yellow crystals. The band gap was calculated from the diffuse reflectance study by UV‐Vis reflectance spectroscopy (Figure 4a, orange color line). From the Tauc plot, the band gap was calculated to be 2.37 eV for the orange crystal (Figure 4b, orange color line). This is higher than the reported MVPb_2_I_6_ material, which is based on the methyl substituted 4‐4’‐bypyridine based hybrid material.^[20]^ It is obvious that the two pyridine rings in the MVPb_2_I_6_ material resides in the same plane, which possesses the central dihedral angle ≈180°, causes smooth conjugation path throughout the system (it is also observed by the red color of the MVPb_2_I_6_ material). But in case of (2‐2’‐DMBPy)_2_Pb_3_I_10_, the central dihedral angle between the two pyridine rings is ≈120°, which somehow hindered the main conjugation inside the organic moiety, causes the higher band gap (Figure 4c). The band gap of the yellow crystal was calculated form the Tauc plot, which was plotted from the diffuse reflectance spectroscopy (Figure 4a, dark yellow color line). It was observed that the yellow powder possesses a band gap of 2.42 eV which is even higher than the orange powder (Figure 4b, dark yellow color line). From the crystal data analysis, it was observed that the central dihedral angle between the two pyridine units of 2‐2’‐DMBPy^2+^ cation is 104°, which closes to perpendicular to each other (Figure 4e). Since the two rings are not in planar, the p‐orbitals are not parallel to each other. This causes the hindrance in conjugation and increase the band gap as well. From the Figure 4d, it is observed that the N_1_−C_1_−C_2_ make one plane and C_1_−C_2_−N_2_ make another plane. The angle between these two planes (dihedral plane) changes significantly leading to the hindrance in conjugation for orange and yellow crystals.

Finally, we'd like to draw an analogy to polymorphism in organic crystals, where different conformers of the same molecule and/or distinct packing arrangements lead to polymorphic crystal structures with unique physical, chemical, and mechanical properties. The stability of these polymorphs is typically governed by intermolecular and intramolecular interactions. Although the two crystals in our study do not share the same composition and thus are not polymorphs in the strictest sense, they can be meaningfully discussed within the context of polymorphism due to the different torsional conformers of the bipyridinium cation. What is particularly interesting in our findings is how a subtle change in the torsion angle can significantly impact the connectivity of the inorganic 1D chains and, subsequently, the optical properties. This induction effect provides a compelling mechanism to tune the properties of organic‐inorganic hybrid materials – an approach that has been relatively unexplored. In line with this, we found one related study by Neilson's group, which used the planarity of organic cations to control the topology of hybrid metal halides.^[27]^

In conclusion, we have synthesized two HOIMHs *i. e*. (2‐2’‐DMBPy)_2_Pb_3_I_10_ (orange crystal) and (2‐2’‐DMBPy)Pb_2_I_6_ (yellow crystal) by reaction between N‐functionalized of 2‐2’‐DMBPy and PbI_2_, where the orange crystal shows higher dihedral angle between the two pyridine rings. This leads to the better conjugation throughout the whole network which causes lowering the band gap compared to the yellow crystal. Thus we anticipate this study will pave the way of fine tuning of ligands inside the hybrid materials and their structural variation.

## Supporting Information Summary

Accession Codes: CCDC **2351398**–**2351399** contains the supplementary crystallographic data for this paper. These data can be obtained free of charge via http://www.ccdc.cam.ac.uk/data_request/cif,or by emailing data_request@ccdc.cam.ac.uk, or by contacting The Cambridge Crystallographic Data Centre 12 Union Road, Cambridge CB2 1EZ, UK; fax: +44 1223 336033.

## Conflict of Interests

The authors declare no conflict of interest.

1

## Supporting information

As a service to our authors and readers, this journal provides supporting information supplied by the authors. Such materials are peer reviewed and may be re‐organized for online delivery, but are not copy‐edited or typeset. Technical support issues arising from supporting information (other than missing files) should be addressed to the authors.

Supporting Information

## Data Availability

The data that support the findings of this study are openly available in CCDC at http://www.ccdc.cam.ac.uk/data_request/cif, reference number 2351398.
