# Supplementary material for: Impact of Dihedral Angle in Conjugated Organic Cation on the Structures and Properties of Organic‐Inorganic Lead Iodides
Source: Chemistry. 2024 Dec 4;31(2):e202402535. doi: 10.1002/chem.202402535 (PMC11724231; doi:10.1002/chem.202402535)
Supplement: Supplementary file 1 — Supporting Information [file CHEM-31-e202402535-s001.pdf]

Chemistry–A European Journal

Supporting Information

Impact of Dihedral Angle in Conjugated Organic Cation on the Structures and Properties of Organic-Inorganic Lead Iodides

Bidhan Chandra Patra, Ruichen Wan, Curtis E. Moore, and Yiying Wu*

Supporting Information

Impact of Dihedral Angle in Conjugated Organic Cation on the Structures and Properties of Organic-Inorganic Lead Iodides

Bidhan Chandra Patra,† Ruichen Wan,† Curtis E. Moore,† and Yiying Wu†*

†Department of Chemistry and Biochemistry, The Ohio State University, Columbus, Ohio 43210, United States.

*Email: wu@chemistry.ohio-state.edu

Materials and Chemicals.

PbI_2 (>99.99%, for perovskite precursor) were purchased from TCI America. 2,2′-bipyridine (98%), iodomethane (contains copper as stabilizer, 99.5%), HI (57 wt %, distilled, stabilized, 99.95%), acetonitrile (ACN, anhydrous, 99.8%), were all purchased from Sigma-Aldrich. All of these chemicals were used as received without further purification.

Structural and Optical Characterizations.

X-ray diffraction on the powder was measured by a Bruker D8 Advance X-ray diffractometer with Cu Kα source and operated at 40 kV, 40 mA. The Pawley refinement was processed using reflex module in material studio software. The single crystal X-ray diffraction studies were carried out on a Bruker Kappa Photon III CPAD diffractometer equipped with Mo K_{α} radiation (λ = 0.71073 Å). A 0.126 x 0.117 x 0.058 mm piece of an orange block ((2-2′-DMBPy)$_2$Pb$_3$I$_{10}$ crystal) and a 0.104 x 0.084 x 0.079 mm piece of a yellow block ((2-2′-DMBPy)Pb$_2$I$_6$ crystal) was mounted on a MiTeGen MicroMount with Paratone 24EX oil. Data were collected in a nitrogen gas stream at 100(2) K using ϕ and ϖ scans. Crystal-to-detector distance was 60 mm and exposure time was 1 second per frame using a scan width of 1.0°. Data collection was 99.7% and 99.2% complete for (2-2′-DMBPy)$_2$Pb$_3$I$_{10}$ (orange) and (2-2′-DMBPy)Pb$_2$I$_6$ (yellow) respectively to 25.00° in θ (0.83Å). A total of 22103 reflections were collected covering the indices, -13<=h<=13, -21<=k<=21, -15<=l<=15 for (2-2′-DMBPy)$_2$Pb$_3$I$_{10}$ and a total of 110946 reflections were collected covering the indices, -21<=h<=21, -19<=k<=20, -22<=l<=22 for (2-2′-DMBPy)Pb$_2$I$_6$ crystals. 4471 reflections were found to be symmetry independent, with a R_{int} of 0.0399 for orange crystal and 5167 reflections were found to be symmetry independent, with a R_{int} of 0.0507 for yellow crystals. The data were integrated using the Bruker SAINT software program and scaled using the SADABS software program. Solution by dual-space method (SHELXT) produced a complete phasing model for refinement.

All nonhydrogen atoms were refined anisotropically by full-matrix least-squares (SHELXL-2014). All hydrogen atoms were placed using a riding model. Their positions were constrained relative to their parent atom using the appropriate HFIX command in SHELXL-2014.

Transmission (%T) and reflectance (%R) spectra of the (2-2'-DMBPy)$_2$Pb$_3$I$_{10}$ and (2-2'-DMBPy)Pb$_2$I$_6$ were measured by the transmission and reflectance modes of an ultraviolet−visible (UV−vis) spectrometer (PerkinElmer Lambda 950). Based on the Kubelka−Munk function, the reflectance can be transferred to absorption coefficient α:

$$\alpha = \frac{(1-\mathrm{R})^2}{2R}$$

where 'α' was also referred to pseudo-absorbance.

Synthesis of 2-2'-dimethyl bipyridinium di-iodide (2-2'-DMBPyI$_2$).

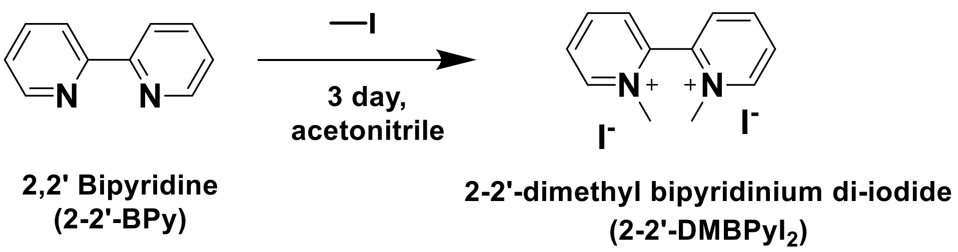

Two millimolar 2,2'-bipyridine and 4 mmol iodomethane were added to 10 mL of acetonitrile. The solution was stirred under 60 °C for 3 days to obtain a greenish yellow colour precipitate. After cooling down, the product was collected by vacuum filtration and washed with acetonitrile. ^{1}H NMR (400 MHz, DMSO-d^6) δ (ppm) = 9.43 (d, J = 8.0 Hz, 2H), 8.9 (t, J = 8.0 Hz, 2H), 8.49 (m, 4H), 4.16 (s, 6H).

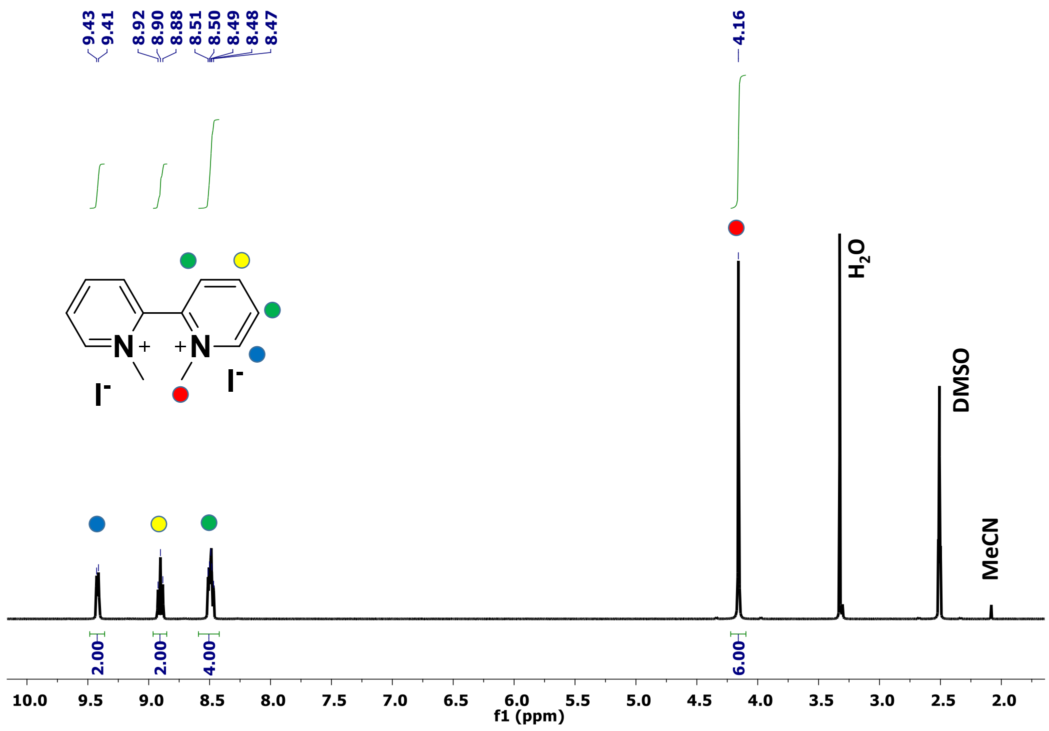

Figure S1: NMR spectra of 2-2'-DMBPyI$_2$

Synthesis of 2-2'-DMBPyPbI.

0.25 mmol PbI$_2$ was dissolved into 1 mL of 57% HI solution to make a clear yellow solution. 0.05 mmol 2-2'-DMBPyI$_2$ was directly added into the above solution and heated for 5 min to dissolve. The solution was cooled down and small crystals were grown gradually (2-2'-DMBPyPbI). 2-2'-DMBPyPbI crystals were grown in yellow and orange color crystals inside the same environment and collected for single-crystal XRD. Orange crystals and yellow crystals were crystalized in the stoichiometric ratio of (2-2'-DMBPy)$_2$Pb$_3$I$_{10}$ and (2-2'-DMBPy)Pb$_2$I$_6$.

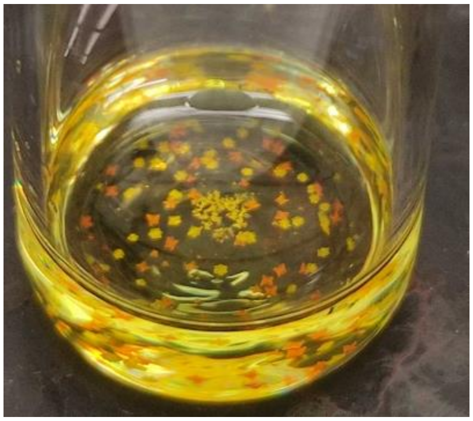

Figure S2: Yellow and orange crystal grown in same environment.

Crystallographic data of orange crystal $(2\text{-}2'\text{-DMBPy})_2Pb_3I_{10}$.

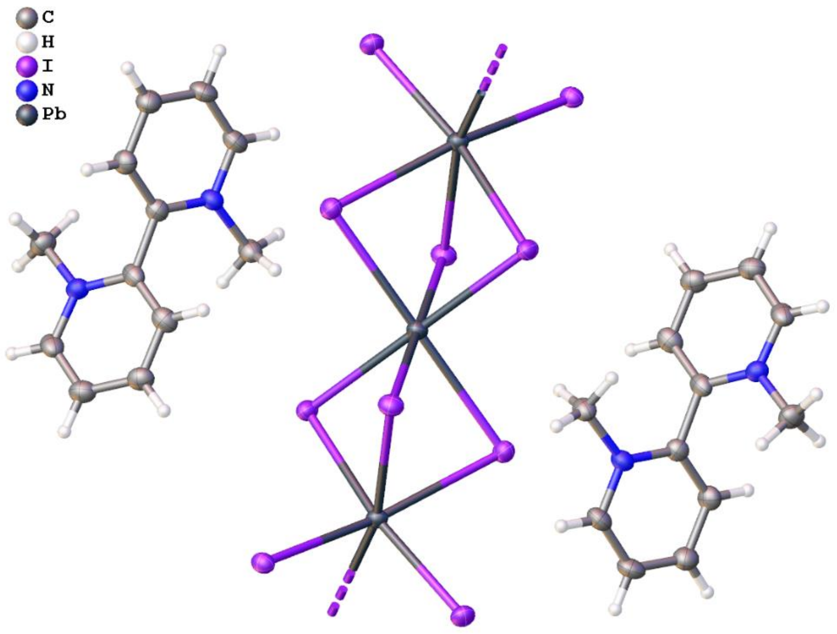

Table S1. Crystal data and structure refinement for Orange crystal $(2\text{-}2'\text{-DMBPy})_2Pb_3I_{10}$.

Empirical formula	C24 H28 I10 N4 Pb3	
Molecular formula	I10 Pb3, 2(C12 H14 N2)	
Formula weight	2263.07	
Temperature	100.0 K	
Wavelength	0.71073 Å	
Crystal system	Monoclinic	
Space group	P 1 21/n 1	
Unit cell dimensions	a = 11.0901(3) Å	α= 90°.
	b = 17.2367(5) Å	β= 112.8130(10)°.
	c = 12.4168(3) Å	γ = 90°.
Volume	2187.88(10) $Å^3$	
Z	2	
Density (calculated)	3.435 Mg/m^3	
Absorption coefficient	18.591 mm^{-1}	
F(000)	1952	
Crystal size	0.126 x 0.117 x 0.058 mm^3	
Crystal color, habit	orange block	

Theta range for data collection	2.136 to 26.387°.
Index ranges	-13<=h<=13, -21<=k<=21, -15<=l<=15
Reflections collected	22103
Independent reflections	4471 [R(int) = 0.0399, R(sigma) = 0.0302]
Completeness to theta = 25.000°	99.7 %
Absorption correction	Semi-empirical from equivalents
Max. and min. transmission	0.0452 and 0.0198
Refinement method	Full-matrix least-squares on F^2
Data / restraints / parameters	4471 / 0 / 200
Goodness-of-fit on F^2	1.195
Final R indices [I>2sigma(I)]	R1 = 0.0214, wR2 = 0.0451
R indices (all data)	R1 = 0.0232, wR2 = 0.0456
Extinction coefficient	0.00032(3)
Largest diff. peak and hole	0.759 and -0.641 e.Å^{-3}

Table S2. Atomic coordinates (x 10^4) and equivalent isotropic displacement parameters (Å^{2}x 10^3) for Orange crystal $(2\text{-}2'\text{-DMBPy})_2Pb_3I_{10}$. U(eq) is defined as one third of the trace of the orthogonalized U^{ij} tensor.

	x	y	z	U(eq)
Pb(1)	888(1)	4758(1)	8607(1)	23(1)
Pb(2)	0	5000	5000	24(1)
I(1)	3379(1)	4099(1)	10454(1)	30(1)
I(2)	859(1)	6186(1)	10247(1)	26(1)
I(3)	1985(1)	5962(1)	7242(1)	29(1)
I(3B)	2130(30)	5801(15)	7390(20)	29(1)
I(4)	1357(1)	3536(1)	6709(1)	27(1)
I(4B)	840(30)	3449(9)	6636(11)	27(1)
I(5)	1984(1)	4968(1)	3706(1)	26(1)
I(5B)	1910(18)	4725(13)	3707(16)	26(1)
N(1)	6513(4)	3035(2)	5581(4)	26(1)
N(2)	5252(4)	3780(2)	7539(4)	25(1)
C(1)	6467(6)	3022(3)	4478(5)	32(1)
C(2)	5344(5)	3208(3)	3545(5)	31(1)
C(3)	4252(5)	3411(3)	3757(5)	31(1)
C(4)	4288(5)	3378(3)	4877(5)	31(1)

C(5)	5426(5)	3201(3)	5791(5)	28(1)
C(6)	5497(5)	3141(3)	7016(4)	26(1)
C(7)	5752(5)	2437(3)	7585(4)	29(1)
C(8)	5785(5)	2386(3)	8724(5)	32(1)
C(9)	5575(5)	3047(3)	9252(5)	33(1)
C(10)	5297(5)	3739(3)	8649(5)	30(1)
C(11)	7797(5)	2883(3)	6540(4)	33(1)
C(12)	4888(5)	4538(3)	6926(5)	30(1)

Table S3. Bond lengths [Å] and angles [°] for Orange crystal ($(2\text{-}2'\text{-DMBPy})_2Pb_3I_{10}$).

Pb(1)-I(1)	3.0443(4)
Pb(1)-I(2)#1	3.2501(4)
Pb(1)-I(2)	3.2020(4)
Pb(1)-I(3)	3.2033(9)
Pb(1)-I(3B)	3.00(3)
Pb(1)-I(4)	3.3496(5)
Pb(1)-I(4B)	3.315(13)
Pb(1)-I(5)#2	3.3943(6)
Pb(1)-I(5B)#2	3.429(18)
Pb(2)-I(3)#2	3.2548(8)
Pb(2)-I(3)	3.2548(8)
Pb(2)-I(3B)	3.29(3)
Pb(2)-I(3B)#2	3.29(3)
Pb(2)-I(4)	3.2657(4)
Pb(2)-I(4)#2	3.2658(4)
Pb(2)-I(4B)#2	3.268(13)
Pb(2)-I(4B)	3.268(13)
Pb(2)-I(5)	3.1895(6)
Pb(2)-I(5)#2	3.1895(6)
Pb(2)-I(5B)#2	3.150(19)
Pb(2)-I(5B)	3.150(19)
N(1)-C(1)	1.351(7)
N(1)-C(5)	1.360(7)
N(1)-C(11)	1.483(6)
N(2)-C(6)	1.357(6)
N(2)-C(10)	1.361(6)
N(2)-C(12)	1.487(6)
C(1)-H(1)	0.9500
C(1)-C(2)	1.370(7)
C(2)-H(2)	0.9500
C(2)-C(3)	1.381(8)
C(3)-H(3)	0.9500
C(3)-C(4)	1.377(7)
C(4)-H(4)	0.9500
C(4)-C(5)	1.365(8)
C(5)-C(6)	1.496(7)
C(6)-C(7)	1.379(7)
C(7)-H(7)	0.9500
C(7)-C(8)	1.402(7)
C(8)-H(8)	0.9500
C(8)-C(9)	1.379(8)
C(9)-H(9)	0.9500
C(9)-C(10)	1.378(8)
C(10)-H(10)	0.9500
C(11)-H(11A)	0.9800
C(11)-H(11B)	0.9800
C(11)-H(11C)	0.9800
C(12)-H(12A)	0.9800

C(12)-H(12B)	0.9800	I(3)#2-Pb(2)-I(4)	98.648(10)
C(12)-H(12C)	0.9800	I(3)#2-Pb(2)-I(4)#2	81.352(10)
		I(3)-Pb(2)-I(4)	81.352(10)
I(1)-Pb(1)-I(2)	92.176(10)	I(3)-Pb(2)-I(4B)#2	92.8(3)
I(1)-Pb(1)-I(2)#1	90.244(10)	I(3)#2-Pb(2)-I(4B)#2	87.2(3)
I(1)-Pb(1)-I(3)	102.730(18)	I(3B)#2-Pb(2)-I(3B)	180.0(8)
I(1)-Pb(1)-I(4)	87.223(16)	I(4)-Pb(2)-I(3B)#2	104.5(4)
I(1)-Pb(1)-I(4B)	93.0(3)	I(4)#2-Pb(2)-I(3B)#2	75.5(4)
I(1)-Pb(1)-I(5)#2	165.65(2)	I(4)-Pb(2)-I(4)#2	180.0
I(1)-Pb(1)-I(5B)#2	171.8(4)	I(4)-Pb(2)-I(4B)#2	170.0(5)
I(2)-Pb(1)-I(2)#1	87.270(10)	I(4)#2-Pb(2)-I(4B)#2	10.0(5)
I(2)-Pb(1)-I(3)	86.449(17)	I(4B)#2-Pb(2)-I(3B)	98.6(4)
I(2)#1-Pb(1)-I(4)	105.91(2)	I(4B)-Pb(2)-I(3B)	81.4(4)
I(2)-Pb(1)-I(4)	166.80(2)	I(4B)#2-Pb(2)-I(4B)	180.0(3)
I(2)#1-Pb(1)-I(4B)	98.0(4)	I(5)-Pb(2)-I(3)	94.01(2)
I(2)-Pb(1)-I(4B)	172.6(2)	I(5)#2-Pb(2)-I(3)#2	94.00(2)
I(2)-Pb(1)-I(5)#2	101.184(16)	I(5)#2-Pb(2)-I(3)	85.99(2)
I(2)#1-Pb(1)-I(5)#2	85.233(15)	I(5)-Pb(2)-I(3)#2	85.99(2)
I(2)-Pb(1)-I(5B)#2	96.0(4)	I(5)-Pb(2)-I(3B)#2	86.1(5)
I(2)#1-Pb(1)-I(5B)#2	89.9(4)	I(5)#2-Pb(2)-I(3B)#2	93.9(5)
I(3)-Pb(1)-I(2)#1	165.778(18)	I(5)-Pb(2)-I(4)#2	85.405(11)
I(3)-Pb(1)-I(4)	80.833(18)	I(5)#2-Pb(2)-I(4)	85.405(11)
I(3)-Pb(1)-I(5)#2	83.489(15)	I(5)-Pb(2)-I(4)	94.594(11)
I(3)-Pb(1)-I(5B)#2	78.1(4)	I(5)#2-Pb(2)-I(4)#2	94.596(11)
I(3B)-Pb(1)-I(1)	98.3(5)	I(5)-Pb(2)-I(4B)#2	77.7(4)
I(3B)-Pb(1)-I(2)	88.8(5)	I(5)#2-Pb(2)-I(4B)#2	102.3(4)
I(3B)-Pb(1)-I(4B)	85.2(6)	I(5)#2-Pb(2)-I(5)	180.0
I(3B)-Pb(1)-I(5B)#2	82.2(5)	I(5B)-Pb(2)-I(3B)	97.7(6)
I(4)-Pb(1)-I(5)#2	80.963(13)	I(5B)#2-Pb(2)-I(3B)	82.3(6)
I(4)-Pb(1)-I(5B)#2	84.9(3)	I(5B)-Pb(2)-I(4B)	96.2(4)
I(4B)-Pb(1)-I(5B)#2	78.9(4)	I(5B)#2-Pb(2)-I(4B)	83.8(4)
I(5)#2-Pb(1)-I(5B)#2	7.2(4)	I(5B)-Pb(2)-I(5B)#2	180.0
I(3)#2-Pb(2)-I(3)	180.00(3)	Pb(1)-I(2)-Pb(1)#1	92.730(10)
I(3)-Pb(2)-I(3B)#2	174.1(4)	Pb(1)-I(3)-Pb(2)	81.539(19)
I(3)#2-Pb(2)-I(3B)#2	5.9(4)	Pb(1)-I(3B)-Pb(2)	84.0(7)
I(3)-Pb(2)-I(4)#2	98.647(10)	Pb(2)-I(4)-Pb(1)	79.204(10)

Pb(2)-I(4B)-Pb(1)	79.7(3)
Pb(2)-I(5)-Pb(1)#2	79.603(12)
Pb(2)-I(5B)-Pb(1)#2	79.6(4)
C(1)-N(1)-C(5)	120.7(4)
C(1)-N(1)-C(11)	117.3(5)
C(5)-N(1)-C(11)	122.0(4)
C(6)-N(2)-C(10)	120.4(4)
C(6)-N(2)-C(12)	122.2(4)
C(10)-N(2)-C(12)	117.4(4)
N(1)-C(1)-H(1)	119.4
N(1)-C(1)-C(2)	121.2(5)
C(2)-C(1)-H(1)	119.4
C(1)-C(2)-H(2)	120.8
C(1)-C(2)-C(3)	118.4(5)
C(3)-C(2)-H(2)	120.8
C(2)-C(3)-H(3)	120.1
C(4)-C(3)-C(2)	119.8(5)
C(4)-C(3)-H(3)	120.1
C(3)-C(4)-H(4)	119.8
C(5)-C(4)-C(3)	120.3(5)
C(5)-C(4)-H(4)	119.8
N(1)-C(5)-C(4)	119.3(5)
N(1)-C(5)-C(6)	118.7(5)
C(4)-C(5)-C(6)	121.9(5)
N(2)-C(6)-C(5)	119.3(4)
N(2)-C(6)-C(7)	120.6(5)
C(7)-C(6)-C(5)	120.1(5)
C(6)-C(7)-H(7)	120.3
C(6)-C(7)-C(8)	119.4(5)
C(8)-C(7)-H(7)	120.3
C(7)-C(8)-H(8)	120.5
C(9)-C(8)-C(7)	119.1(5)
C(9)-C(8)-H(8)	120.5
C(8)-C(9)-H(9)	120.0
C(10)-C(9)-C(8)	119.9(5)
C(10)-C(9)-H(9)	120.0
N(2)-C(10)-C(9)	120.5(5)
N(2)-C(10)-H(10)	119.7
C(9)-C(10)-H(10)	119.7
N(1)-C(11)-H(11A)	109.5
N(1)-C(11)-H(11B)	109.5
N(1)-C(11)-H(11C)	109.5
H(11A)-C(11)-H(11B)	109.5
H(11A)-C(11)-H(11C)	109.5
H(11B)-C(11)-H(11C)	109.5
N(2)-C(12)-H(12A)	109.5
N(2)-C(12)-H(12B)	109.5
N(2)-C(12)-H(12C)	109.5
H(12A)-C(12)-H(12B)	109.5
H(12A)-C(12)-H(12C)	109.5
H(12B)-C(12)-H(12C)	109.5

Symmetry transformations used to generate equivalent atoms:

#1 -x,-y+1,-z+2   #2 -x,-y+1,-z+1

Table S4. Anisotropic displacement parameters ($Å^2$x 10^3) for Wu_Bidhan_Orange. The anisotropic displacement factor exponent takes the form: $-2p^2[h^2 a^{*2}U^{11} + ... + 2 h k a^* b^* U^{12}]$

	U^{11}	U^{22}	U^{33}	U^{23}	U^{13}	U^{12}
Pb(1)	25(1)	24(1)	19(1)	1(1)	7(1)	0(1)
Pb(2)	25(1)	26(1)	20(1)	1(1)	9(1)	1(1)
I(1)	29(1)	33(1)	23(1)	3(1)	6(1)	4(1)
I(2)	32(1)	24(1)	23(1)	-2(1)	12(1)	-3(1)
I(3)	32(1)	28(1)	28(1)	1(1)	13(1)	-5(1)
I(3B)	32(1)	28(1)	28(1)	1(1)	13(1)	-5(1)
I(4)	30(1)	25(1)	24(1)	-1(1)	8(1)	2(1)
I(4B)	30(1)	25(1)	24(1)	-1(1)	8(1)	2(1)
I(5)	24(1)	30(1)	25(1)	5(1)	10(1)	4(1)
I(5B)	24(1)	30(1)	25(1)	5(1)	10(1)	4(1)
N(1)	29(2)	25(2)	23(2)	1(2)	8(2)	1(2)
N(2)	29(2)	26(2)	23(2)	-1(2)	12(2)	-3(2)
C(1)	42(3)	25(3)	37(3)	5(2)	25(3)	0(2)
C(2)	37(3)	30(3)	26(3)	0(2)	10(2)	-5(2)
C(3)	35(3)	29(3)	25(3)	-3(2)	7(2)	-5(2)
C(4)	34(3)	29(3)	31(3)	-7(2)	15(2)	-9(2)
C(5)	39(3)	18(3)	31(3)	-2(2)	21(2)	-2(2)
C(6)	25(3)	26(3)	25(2)	-3(2)	8(2)	-4(2)
C(7)	35(3)	24(3)	29(3)	-4(2)	12(2)	-2(2)
C(8)	34(3)	32(3)	32(3)	3(2)	15(2)	-2(2)
C(9)	39(3)	39(3)	24(3)	1(2)	16(2)	-6(3)
C(10)	33(3)	36(3)	26(3)	-4(2)	17(2)	-4(2)
C(11)	30(3)	34(3)	28(3)	0(2)	6(2)	0(2)
C(12)	38(3)	24(3)	33(3)	2(2)	20(2)	-2(2)

Table S5. Hydrogen coordinates (x 10^4) and isotropic displacement parameters ($Å^2$x 10^{3}) for Orange crystal ($(2\text{-}2'\text{-DMBPy})_2Pb_3I_{10}$).

	x	y	z	U(eq)
H(1)	7227	2880	4346	38
H(2)	5318	3198	2771	38
H(3)	3477	3574	3131	37
H(4)	3518	3479	5014	37
H(7)	5904	1989	7210	35
H(8)	5950	1904	9125	39
H(9)	5622	3026	10032	39
H(10)	5136	4191	9010	36
H(11A)	7855	3173	7237	49
H(11B)	8500	3049	6299	49
H(11C)	7883	2327	6717	49
H(12A)	3952	4539	6433	45
H(12B)	5391	4618	6438	45
H(12C)	5082	4956	7503	45

Crystallographic data of yellow crystal (2-2'-DMBPy)Pb_2I_6.

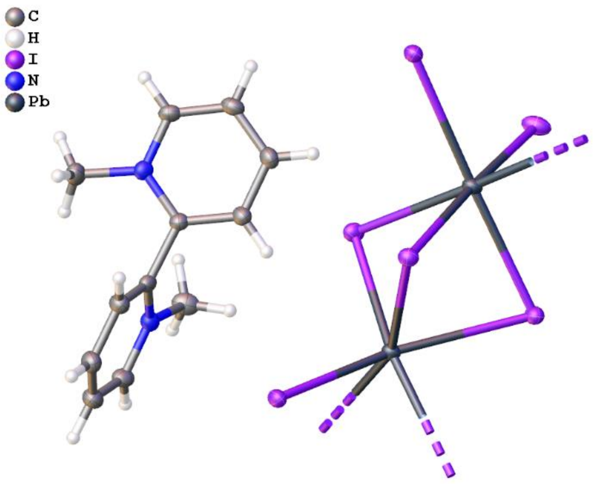

Table S6. Crystal data and structure refinement for Yellow crystal ((2-2'-DMBPy)Pb_2I_6).

Empirical formula	C12 H14 I6 N2 Pb2	
Molecular formula	I6 Pb2, C12 H14 N2	
Formula weight	1362.03	
Temperature	100.0 K	
Wavelength	0.71073 Å	
Crystal system	Orthorhombic	
Space group	Pbca	
Unit cell dimensions	a = 17.5748(4) Å	α= 90°.
	b = 16.0015(3) Å	β= 90°.
	c = 18.0671(3) Å	γ = 90°.
Volume	5080.89(17) $Å^3$	
Z	8	
Density (calculated)	3.561 Mg/m^3	
Absorption coefficient	20.526 mm^{-1}	
F(000)	4656	
Crystal size	0.104 x 0.084 x 0.079 mm^3	
Crystal color, habit	yellow block	
Theta range for data collection	2.057 to 26.397°.	
Index ranges	-21<=h<=21, -19<=k<=20, -22<=l<=22	
Reflections collected	110946	
Independent reflections	5167 [R(int) = 0.0507, R(sigma) = 0.0190]	
Completeness to theta = 25.000°	99.2 %	
Absorption correction	Semi-empirical from equivalents	
Max. and min. transmission	0.0932 and 0.0478	

Refinement method	Full-matrix least-squares on F^2
Data / restraints / parameters	5167 / 0 / 205
Goodness-of-fit on F^2	1.138
Final R indices [I>2sigma(I)]	R1 = 0.0154, wR2 = 0.0316
R indices (all data)	R1 = 0.0188, wR2 = 0.0328
Extinction coefficient	n/a
Largest diff. peak and hole	0.695 and -0.621 e.Å^{-3}

Table S7. Atomic coordinates (x 10^4) and equivalent isotropic displacement parameters (Å^{2}x 10^3) for Yellow crystal ((2-2'-DMBPy)Pb_2I_6). U(eq) is defined as one third of the trace of the orthogonalized U^{ij} tensor.

	x	y	z	U(eq)
Pb(1)	7466(1)	7351(1)	6519(1)	18(1)
Pb(2)	7508(1)	4856(1)	6518(1)	19(1)
I(1)	6437(1)	8604(1)	5552(1)	21(1)
I(2)	7163(1)	6138(1)	5157(1)	20(1)
I(3)	8873(1)	6069(1)	6911(1)	22(1)
I(4)	6429(1)	6070(1)	7464(1)	22(1)
I(5)	6160(1)	3587(1)	6027(1)	21(1)
I(6)	7906(2)	3705(1)	7853(1)	28(1)
I(6B)	7691(11)	3709(4)	7869(4)	28(1)
N(1)	4620(2)	8357(2)	6530(2)	22(1)
N(2)	3758(2)	6562(2)	6019(2)	21(1)
C(1)	4615(2)	9203(2)	6532(2)	25(1)

C(2)	4385(2)	9648(2)	5924(2)	29(1)
C(3)	4177(2)	9232(2)	5293(2)	27(1)
C(4)	4187(2)	8358(2)	5291(2)	25(1)
C(5)	4395(2)	7926(2)	5918(2)	21(1)
C(6)	4424(2)	6991(2)	5934(2)	20(1)
C(7)	5093(2)	6564(2)	5832(2)	23(1)
C(8)	5096(2)	5695(2)	5834(2)	25(1)
C(9)	4422(2)	5272(2)	5930(2)	25(1)
C(10)	3756(2)	5714(2)	6017(2)	22(1)
C(11)	4927(2)	7920(2)	7189(2)	30(1)
C(12)	3025(2)	6996(2)	6087(2)	28(1)

Table S8. Bond lengths [Å] and angles [°] for Yellow crystal ((2-2'-DMBPy)Pb_2I_6).

Pb(1)-I(1)	3.2158(2)	Pb(2)-I(4)	3.2093(2)
Pb(1)-I(2)	3.1779(2)	Pb(2)-I(5)	3.2426(2)
Pb(1)-I(3)	3.2899(2)	Pb(2)-I(6)	3.1148(9)
Pb(1)-I(4)	3.2304(2)	Pb(2)-I(6B)	3.071(7)
Pb(1)-I(5)#1	3.2469(2)	N(1)-C(1)	1.353(4)
Pb(1)-I(6)#1	3.3072(8)	N(1)-C(5)	1.362(4)
Pb(1)-I(6B)#1	3.279(7)	N(1)-C(11)	1.483(4)
Pb(2)-I(1)#2	3.2399(2)	N(2)-C(6)	1.365(4)
Pb(2)-I(2)	3.2584(2)	N(2)-C(10)	1.357(4)
Pb(2)-I(3)	3.1679(3)	N(2)-C(12)	1.469(4)

C(1)-H(1)	0.9500
C(1)-C(2)	1.371(5)
C(2)-H(2)	0.9500
C(2)-C(3)	1.369(5)
C(3)-H(3)	0.9500
C(3)-C(4)	1.398(5)
C(4)-H(4)	0.9500
C(4)-C(5)	1.376(5)
C(5)-C(6)	1.498(4)
C(6)-C(7)	1.372(5)
C(7)-H(7)	0.9500
C(7)-C(8)	1.390(5)
C(8)-H(8)	0.9500
C(8)-C(9)	1.374(5)
C(9)-H(9)	0.9500
C(9)-C(10)	1.376(5)
C(10)-H(10)	0.9500
C(11)-H(11A)	0.9800
C(11)-H(11B)	0.9800
C(11)-H(11C)	0.9800
C(12)-H(12A)	0.9800
C(12)-H(12B)	0.9800
C(12)-H(12C)	0.9800
I(1)-Pb(1)-I(3)	158.162(7)
I(1)-Pb(1)-I(4)	111.456(7)
I(1)-Pb(1)-I(5)#1	83.672(6)
I(1)-Pb(1)-I(6)#1	82.89(4)
I(1)-Pb(1)-I(6B)#1	86.8(2)
I(2)-Pb(1)-I(1)	82.324(6)
I(2)-Pb(1)-I(3)	84.934(6)
I(2)-Pb(1)-I(4)	85.874(6)
I(2)-Pb(1)-I(5)#1	106.554(7)
I(2)-Pb(1)-I(6)#1	158.67(6)
I(2)-Pb(1)-I(6B)#1	165.2(3)
I(3)-Pb(1)-I(6)#1	113.58(5)
I(4)-Pb(1)-I(3)	85.121(6)
I(4)-Pb(1)-I(5)#1	161.947(7)
I(4)-Pb(1)-I(6)#1	85.32(3)
I(4)-Pb(1)-I(6B)#1	88.8(2)
I(5)#1-Pb(1)-I(3)	83.058(6)
I(5)#1-Pb(1)-I(6)#1	86.96(5)
I(5)#1-Pb(1)-I(6B)#1	82.1(3)
I(6B)#1-Pb(1)-I(3)	108.4(3)
I(1)#2-Pb(2)-I(2)	95.123(6)
I(1)#2-Pb(2)-I(5)	83.359(6)
I(3)-Pb(2)-I(1)#2	93.780(7)
I(3)-Pb(2)-I(2)	85.620(6)
I(3)-Pb(2)-I(4)	87.508(6)
I(3)-Pb(2)-I(5)	176.713(7)

I(4)-Pb(2)-I(1)#2	178.710(7)	C(10)-N(2)-C(12)	118.0(3)
I(4)-Pb(2)-I(2)	84.898(6)	N(1)-C(1)-H(1)	119.4
I(4)-Pb(2)-I(5)	95.352(6)	N(1)-C(1)-C(2)	121.2(3)
I(5)-Pb(2)-I(2)	93.009(6)	C(2)-C(1)-H(1)	119.4
I(6)-Pb(2)-I(1)#2	85.58(4)	C(1)-C(2)-H(2)	120.2
I(6)-Pb(2)-I(2)	176.63(4)	C(3)-C(2)-C(1)	119.6(3)
I(6)-Pb(2)-I(3)	91.04(5)	C(3)-C(2)-H(2)	120.2
I(6)-Pb(2)-I(4)	94.47(4)	C(2)-C(3)-H(3)	120.5
I(6)-Pb(2)-I(5)	90.35(5)	C(2)-C(3)-C(4)	118.9(3)
I(6B)-Pb(2)-I(2)	174.5(3)	C(4)-C(3)-H(3)	120.5
I(6B)-Pb(2)-I(3)	96.2(3)	C(3)-C(4)-H(4)	119.9
I(6B)-Pb(2)-I(4)	90.0(2)	C(5)-C(4)-C(3)	120.3(3)
I(6B)-Pb(2)-I(5)	85.4(3)	C(5)-C(4)-H(4)	119.9
Pb(1)-I(1)-Pb(2)#1	76.766(6)	N(1)-C(5)-C(4)	119.4(3)
Pb(1)-I(2)-Pb(2)	76.682(6)	N(1)-C(5)-C(6)	118.7(3)
Pb(2)-I(3)-Pb(1)	76.365(6)	C(4)-C(5)-C(6)	121.8(3)
Pb(2)-I(4)-Pb(1)	76.645(6)	N(2)-C(6)-C(5)	118.4(3)
Pb(2)-I(5)-Pb(1)#2	76.295(6)	N(2)-C(6)-C(7)	119.9(3)
Pb(2)-I(6)-Pb(1)#2	77.18(2)	C(7)-C(6)-C(5)	121.7(3)
Pb(2)-I(6B)-Pb(1)#2	78.21(15)	C(6)-C(7)-H(7)	119.9
C(1)-N(1)-C(5)	120.5(3)	C(6)-C(7)-C(8)	120.1(3)
C(1)-N(1)-C(11)	118.2(3)	C(8)-C(7)-H(7)	119.9
C(5)-N(1)-C(11)	121.2(3)	C(7)-C(8)-H(8)	120.4
C(6)-N(2)-C(12)	121.6(3)	C(9)-C(8)-C(7)	119.2(3)
C(10)-N(2)-C(6)	120.3(3)	C(9)-C(8)-H(8)	120.4

C(8)-C(9)-H(9)	120.2
C(8)-C(9)-C(10)	119.6(3)
C(10)-C(9)-H(9)	120.2
N(2)-C(10)-C(9)	120.8(3)
N(2)-C(10)-H(10)	119.6
C(9)-C(10)-H(10)	119.6
N(1)-C(11)-H(11A)	109.5
N(1)-C(11)-H(11B)	109.5
N(1)-C(11)-H(11C)	109.5
H(11A)-C(11)-H(11B)	109.5
H(11A)-C(11)-H(11C)	109.5
H(11B)-C(11)-H(11C)	109.5
N(2)-C(12)-H(12A)	109.5
N(2)-C(12)-H(12B)	109.5
N(2)-C(12)-H(12C)	109.5
H(12A)-C(12)-H(12B)	109.5
H(12A)-C(12)-H(12C)	109.5
H(12B)-C(12)-H(12C)	109.5

____

Symmetry transformations used to generate equivalent atoms:

#1 -x+3/2,y+1/2,z   #2 -x+3/2,y-1/2,z

Table S9. Anisotropic displacement parameters ($Å^2$x 10^3) for Yellow ((2-2'-DMBPy)Pb_2I_6). The anisotropic displacement factor exponent takes the form: -2p^2[h^2 a*^{2}U^{11} + ... + 2 h k a* b* U^{12}]

	U^{11}	U^{22}	U^{33}	U^{23}	U^{13}	U^{12}
Pb(1)	20(1)	14(1)	20(1)	1(1)	0(1)	0(1)
Pb(2)	23(1)	14(1)	21(1)	1(1)	0(1)	-1(1)
I(1)	20(1)	17(1)	28(1)	1(1)	-3(1)	1(1)
I(2)	23(1)	19(1)	19(1)	1(1)	0(1)	-1(1)
I(3)	21(1)	18(1)	26(1)	0(1)	-4(1)	0(1)
I(4)	23(1)	21(1)	23(1)	0(1)	4(1)	-2(1)
I(5)	20(1)	16(1)	27(1)	1(1)	-1(1)	0(1)
I(6)	47(1)	19(1)	19(1)	2(1)	-4(1)	-5(1)
I(6B)	47(1)	19(1)	19(1)	2(1)	-4(1)	-5(1)
N(1)	20(2)	20(1)	26(2)	-1(1)	3(1)	1(1)
N(2)	20(2)	21(1)	21(1)	-1(1)	3(1)	-1(1)
C(1)	26(2)	20(2)	28(2)	-6(1)	4(2)	-2(2)
C(2)	26(2)	20(2)	40(2)	-3(2)	1(2)	2(2)
C(3)	24(2)	24(2)	35(2)	1(2)	-4(2)	3(2)
C(4)	23(2)	20(2)	32(2)	0(1)	-2(2)	1(1)
C(5)	15(2)	19(2)	30(2)	-3(1)	4(1)	-1(1)
C(6)	19(2)	17(2)	23(2)	-1(1)	-1(1)	-2(1)
C(7)	21(2)	19(2)	29(2)	0(1)	-4(1)	1(1)

C(8)	24(2)	21(2)	31(2)	0(1)	-3(2)	5(1)
C(9)	31(2)	16(2)	28(2)	0(1)	-3(2)	-1(1)
C(10)	25(2)	18(2)	23(2)	2(1)	0(1)	-5(1)
C(11)	41(2)	27(2)	21(2)	1(2)	1(2)	-3(2)
C(12)	22(2)	26(2)	38(2)	4(2)	6(2)	2(2)

Table S10. Hydrogen coordinates (x 10^4) and isotropic displacement parameters ($Å^2$x 10^3) for Yellow crystal ((2-2'-DMBPy)Pb_2I_6).

	x	y	z	U(eq)
H(1)	4774	9494	6964	30
H(2)	4370	10241	5939	34
H(3)	4028	9533	4863	33
H(4)	4049	8062	4856	30
H(7)	5554	6862	5760	27
H(8)	5559	5397	5771	30
H(9)	4416	4679	5936	30
H(10)	3290	5422	6076	26
H(11A)	4618	7424	7293	44
H(11B)	4914	8296	7616	44
H(11C)	5454	7749	7093	44
H(12A)	3093	7510	6375	43
H(12B)	2659	6631	6339	43
H(12C)	2833	7136	5593	43
